# The Book World of Medicine and Science

**Published:** 1897-04-17

**Authors:** 


### The Book World of Medicine and Science.

Wasted Records of Disease. By Charles E. Paget, Lecturer on Health to the Owen's College, Examiner in Public Health in the Victoria University, Medical Officer of Health for the County Borough of Salford. (Edward Arnold, London and New York, 1897. Price 2s. 6d., 90 pages.)

This book gives in the first instance a historical sketch of the individual, semi-private, or local efforts made during the present century to secure or establish permanent systems of disease registration. It is pointed out how in all cases failure of these to survive was due to the fact that they depended on voluntary contributions and on the individual efforts of a few persons, and that they had not official support nor organisation on such a scale as was necessary to their maintenance. It is shown that mortality statistics are utterly inadequate data from which to infer either the prevalence of disease or the general sanitary state of the people. A short sketch follows of the legislative recognition of the necessity of disease registration which resulted in the appointment of medical officers of health. The inadequacy of this enterprise as dealing with certain infectious or zymotic diseases is evident. The attitude of the Government with regard to this question is one of half-hearted admission of the value of any system of disease registration, a partial recognition of the value of early information with regard to the prevalence of infectious diseases, and barely any recognition whatever of the value of properly tabulated records being issued from the materials in its possession. As regards Poor Law returns they also cumber the store-rooms of the Boards of Guardians. They are not used for general information as they should be, but wasted by the Guardians for want of direction, and by the State through indifference. The author suggests as steps in the direction of more complete registration that the notification of infectious diseases should be made compulsory throughout the provinces. A weekly return should be made of these. In addition to periodic Poor Law returns statistics from hospitals, friendly societies, post office and police records should be obtained, all such returns being in the province of the Registrar-General. The book indicates very clearly the present great waste of material already in the possession of the State.

Sight Testing for the G.P. By F. Davidson. (London: A. La Riviere.)

This little pamphlet, which has been forwarded to us, is a description of the method of examining the refraction of the eye by aid of the apparatus devised by the author. Of the method we have already given an account, and we have only to add that this brochure gives a simple description of the proceeding which seems well fitted for its purpose. The mystic letters G.P., be it understood, represent "general practitioner," and lest any disrespect should be imagined the author states that "a well-known general practitioner" suggested the abbreviation.

